# Intergenerational altruism and climate policy preferences

**DOI:** 10.1093/pnasnexus/pgae105

**Published:** 2024-03-13

**Authors:** Gustav Agneman, Sofia Henriks, Hanna Bäck, Emma Renström

**Affiliations:** Department of Economics, Norwegian University of Science and Technology, 7030, Trondheim, Norway; Department of Economics, Gothenburg University, 40530, Gothenburg, Sweden; Department of Political Science, Lund University, 22362, Lund, Sweden; Department of Psychology, Kristianstad University, 29139, Kristianstad, Sweden

**Keywords:** intergenerational altruism, climate policy preferences, survey experiment

## Abstract

Climate mitigation constitutes an intergenerational moral dilemma; the decisions we make today will inevitably shape the prospects for generations to come. Yet, we still know little about the relationship between intergenerational altruism (IGA)—our concerns for the well-being of future generations—and support for costly climate mitigation policies. In this study, we present an approach to measuring IGA through an intergenerational dilemma, where participants allocate resources across generations. First, we describe how IGA depends on the temporal (social) distance between generations and demonstrate robust correlations between IGA and support for several climate policies. Then, we leverage randomized participation in the intergenerational dilemma to show that it causally increases climate policy support, an effect we attribute to higher worries about human-induced climate change among treated subjects. An exploratory heterogeneity analysis suggests that the impact of the intergenerational dilemma is primarily driven by female and nonbinary participants. In sum, this study presents both a novel measurement strategy and robust evidence of a malleable moral basis of climate policy preferences.

**Significance Statement**

Although intergenerational altruism (IGA) is at the heart of collective decision-making of paramount importance, such as climate mitigation efforts, its empirical measurement and role remain underexplored. This study presents an experiment, termed intergenerational dilemma, that allows us to capture variation in IGA and estimate parameters of a theoretical behavioral model that describes its shape. Furthermore, we provide evidence of a strong statistical link between IGA and climate policy support, and show that random assignment to partake in the intergenerational dilemma increases proenviromental political preferences. In sum, this paper presents methodological advances allowing the empirical measurement of a first-order predictor of climate policy preferences, namely IGA.

## Introduction

Governments rely on a parameter known as the social discount rate to compute the present value of long-term projects, which in turn determines how we allocate resources between conventional and green investment alternatives (1). This crucial parameter thus regulates how we prioritize the needs of the present generation relative to those of future generations, an inherently moral tradeoff (2, 3). For instance, this tradeoff is at the heart of climate mitigation policies like carbon taxes, which increase the relative prices of carbon-based consumer goods but mitigate global warming that disproportionally will harm future generations (4, 5). Despite the moral nature of this intergenerational dilemma, we know little about how the general public intrinsically values the well-being of future generations—often referred to as *intergenerational altruism* (IGA) (6)—and how this influences their preferences for climate mitigation policies.

In this study, we both derive a measure of IGA and demonstrate its critical role in shaping climate policy preferences (CPP). The study builds on a nationwide survey in Sweden, where a random subset of the participants took part in an experiment, henceforth referred to as *intergenerational dilemma*, before responding to a series of policy vignettes probing support for costly climate mitigation policies. The experiment consists of two components: (i) a text informing participants about their respective projected number of descendants over the next 250 years and (ii) a resource allocation task in which participants distribute fictive resources across generations.

First, we use the data from the resource allocation task to estimate a behavioral model that structurally describes how the level of altruism dissipates with the temporal (social) distance between generations, i.e. the shape of IGA. Second, we document a strong statistical association between IGA and CPP, which is unaffected by the inclusion of a range of controls. Third, we leverage randomized participation in the intergenerational dilemma to investigate its causal effect on climate preferences, and document a positive influence on support for four different climate policies. Finally, in an exploratory gender-decomposition analysis, we find that women and nonbinary people are affected by participation in the intergenerational dilemma, whereas men are not.

Our study adds to a rich literature concerned with how moral attitudes shape preferences for costly climate action (see, e.g. Refs. (7–11)). This strand of research has, inter alia, linked climate preferences with universal moral values understood as ethical behavior that transcends geographical borders (see, e.g. Ref. (12)). However, the extent to which *temporal* moral deliberations shape CPP among the general public remains underexplored, despite the (very) long-term nature of climate mitigation efforts.^a^

The fact that future generations have not yet come into existence constitutes an apparent obstacle to empirical research on intergenerational moral boundaries. Consequently, researchers have often turned to economic games to simulate cross-generational deliberations (15–21). However, the stylized nature of these games—for instance, in Refs. (16, 18), future generations are “role-played” by subsets of participants—increases the risk of social desirability bias and arguably reduces the real-life generalizability of the findings. In this paper, we leverage randomized participation in an intergenerational dilemma to support a causal interpretation of moral deliberations on CPP. The decoupling of experimental treatment and outcome mitigates the risks of social desirability bias, and the use of several vignettes ensures a valid measurement of CPP.

Our work relates closely to growing bodies of research on how legacy motivations (22–32), a sense of responsibility to protect future generations (33–37), and generativity (38–44) influence proenvironmental outcomes. We add to this important scholarship by providing a novel approach to both measuring and activating IGA. In addition, our paper adds suggestive evidence of a mediator (climatic impact worries) and a moderator (gender) linking intertemporal moral concerns with climate attitudes. Understanding the current lack of climate mitigation efforts as a consequence of social distance to future generations, akin to ingroup–outgroup dynamics in other social domains, e.g. Refs. (45, 46), opens up new avenues for promoting public support for more ambitious policies, inter alia, through attempts at expanding our moral horizon temporally (10). More generally, the results lend support to the notion that norm-related nudges can yield substantial behavioral effects (47).

## Theory

Human morality is not calibrated to effectively combat the risks of climate change. As one of six moral deficiencies, Markowitz and Shariff (10) identified moral parochialism—our lack of concern for the welfare of “outgroups”—as a key impediment to more ambitious climate policies. In a sense, the ultimate outgroup is (distant) future generations, since they are complete strangers to us. Moreover, the temporal distance to future generations is such that they “can neither help us out of reciprocity for our actions nor harm us out of retribution for our inactions” (10, p. 246).

Still, most people appear to intrinsically value the welfare of future generations (48), a preference that we label IGA.^b^ Essentially, IGA determines how we navigate *intergenerational dilemmas*, where the present generation has unilateral decision-making power while being completely unaffected by future consequences of their choice (49). As such, this operationalization refers to pure altruism, in the sense that it encompasses the *intrinsic* value we place on the welfare of future generations (6). In the words of Wade-Benzoni and Tost (49, p. 166), it captures “the extent to which members of present generations are willing to sacrifice their own self-interest for the benefit of future others in the absence of economic or material incentives [⋯]”.

In line with Wade-Benzoni and Tost (49), we posit that the level of altruism should be inversely related to the temporal distance between generations. The reason is that perceived social distance, which depresses other-regarding behaviors (50), is expected to increase as generations become more distant in time. Moreover, building on the theoretical depiction of IGA presented in Ref. (6), we argue that intergenerational preferences are characterized by a present bias. That is, the present generation is “more reluctant to transfer consumption from itself to the next generation, than from any future generation to a later one” (6, p. 1,177). Accordingly, we can characterize the utility we derive from consumption today and from the stream of future generations’ consumption through a quasihyperbolic discount function,^c^ which includes both a social discount rate (*γ*) and a parameter that captures present bias (*β*).^d^


(1) $$U(c_{t})=\alpha \times c_{0}+\beta \times \sum_{t}^{\infty }\frac{c_{t}}{\left(1+\gamma \times t\right)}.$$

The parameters of the utility function are arguably subjective (3), a feature that entails two important consequences. First, although social discounting is a general phenomenon (51), the extent of discounting (and, hence, intergenerational altruism) is expected to vary significantly between and within groups (52). Therefore, the first objective of this study is to characterize both regularity and variation in IGA within a sample of conationals. Second, given that the level of IGA is inversely related to *perceived* social distance, measures that reduce this perceived distance should promote behaviors that benefit future generations. In line with this proposition, Wade-Benzoni and Tost (49, p. 170), claimed that “affinity and identification with future others can diminish interpersonal distance, decrease social discounting, limit egocentric biases, and enhance intergenerational beneficence”. The second objective of this study is to demonstrate that engagement with (the needs of) future generations can increase proenvironmental political preferences. In what follows, we present the research design employed to achieve these objectives.

## Research design

**Fig. 1.**
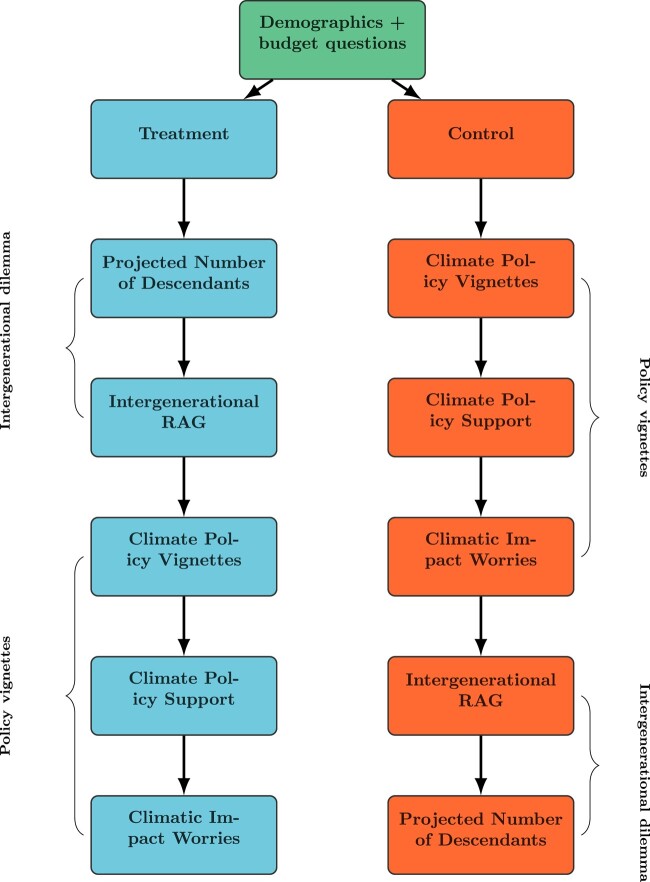
Flowchart presenting the order of survey sections.

To investigate the link between IGA and CPP, we collected survey data from a sample of 1,615 adults in Sweden, a country typically characterized as a front-runner in the green transition (53). The survey included a randomized element in which (treated) participants took part in an intergenerational dilemma. The dilemma consisted of two components. First, participants were asked about their number of children (current and planned) and informed about their estimated number of descendants over the next 250 years.^e^ Then, the same participants were asked to divide a fixed amount of resources between the present and future generations (today, the year 2100, the year 2300, and the year 2500). Finally, they responded to four separate policy vignettes probing support for different climate policies. Conversely, the control group first answered the policy vignettes and then completed the intergenerational dilemma. We visually present the research design in Fig. 1 and provide a more in-depth description in Section A of the supplementary material. In Section B of the supplementary material, we document the exact wording of survey questions and show that randomization yielded balanced subsamples.

The outcome of main interest is CPP. We presented participants with four distinct policy proposals (vignettes), each of which would reduce consumption-induced carbon emissions in Sweden, and measured participants’ support for the different policies on a 10-point scale. The policies would also increase the private consumption costs of respective goods (aviation, food, fuel, and apparel). We calculated the participant-specific rise in consumption costs by first eliciting participants’ annual budgetary spending on related goods and then multiplied these numbers by markup factors specific to each policy scenario. The markup factors were randomly drawn from three predefined levels (low, medium, and high markups). The personalized climate policy costs are described in more detail in Section A2 of the supplementary material.

A secondary outcome, which is analyzed to shed light on *why* the intergenerational dilemma might affect CPP, is climatic impact worries. In these questions, we asked participants to rate their degree of concern about the climatic impacts of (i) their own and (ii) other Swedish citizens’ consumption of respective goods. The survey questions were included after the question probing policy support in order to mitigate the risk that the main outcome (policy support) is contaminated by experimenter demand bias.

The exact wording of each policy vignette and the corresponding questions on support and climatic impact worries are outlined in detail in Section A of the supplementary material.^f^ Note that the ordering of sections implies that survey sections further down the pipeline are “primed” for both the treatment and control groups. Since participants in the control group complete climate policy vignettes before the intergenerational dilemma, they may undertake the resource allocation game with climate change in mind. This means that we can neither test whether informing participants about their projected number of descendants leads to an increase in IGA, nor explore heterogeneous treatment effects on the basis of differences in baseline IGA. Instead, we rely on reduced-form specifications to document (i) how intergenerational altruism relates to CPP and (ii) the causal effect of participating in the intergenerational dilemma. Specifically, we estimate variants of the following models.

Section 4.1:


(2) $${\text{Resource Allocation}}_{\mathrm{id}}=f({\text{Generation}}_{d})+\varepsilon_{\mathrm{id}},$$

which describes how individuals (*i*) allocate resources to the different generations based on their temporal distance (*d*).

Section 4.2:


(3) $${\text{CPP}}_{i}=\beta_{0}+\beta_{1}\times {\text{Intergenerational Altruism}}_{i}+\gamma^{⊤}X_{i}+\varepsilon_{i},$$

which regresses individuals’ (*i*) CPP on their intergenerational altruism and a set of controls (X).

Section 4.3:


(4) $${\text{Y}}_{\text{i}k}=\beta_{0k}+\beta_{1k}\times {\text{Intergenerational Dilemma}}_{\;j}+\varepsilon_{\text{i}k},$$

which estimates the effect of participating in the intergenerational dilemma (*j*) on the *k*th outcome (CPP/climatic impact worries).

Section 4.4:


(5) $${\text{Y}}_{\text{i}kg}=\beta_{0k\text{g}}+\beta_{1kg}\times {\text{Intergenerational Dilemma}}_{\;j}+\varepsilon_{\text{i}kg},$$

which estimates the effect of participating in the intergenerational dilemma (*j*) on the *k*th outcome (CPP/climatic impact worries), separately for different genders (*g*).

## Results

In what follows, we first undertake a descriptive exercise to document empircally the shape of IGA (Section 4.1). Next, we investigate the statistical link between IGA and CPP (Section 4.2). Then, we leverage the random exposure to the intergenerational dilemma to study whether moral deliberations can causally influence CPP as well as worries about the climatic consequences of consumption (Section 4.3). Finally, we gender-decompose the experimental analysis and show that the baseline results are driven by women and nonbinary participants (Section 4.4).

### Intergenerational altruism

The resource allocation game embedded in the intergenerational dilemma provides a measure of IGA (see Ref. (55) for a similar approach). We argue that a more even spread of resources across generations indicates a higher level of IGA compared to a distribution that disproportionately favors the present generation. This way of operationalizing IGA mirrors how altruism has been theorized in other contexts (e.g. in Ref. (46), altruism diminishes as social distance increases).

**Fig. 2.**
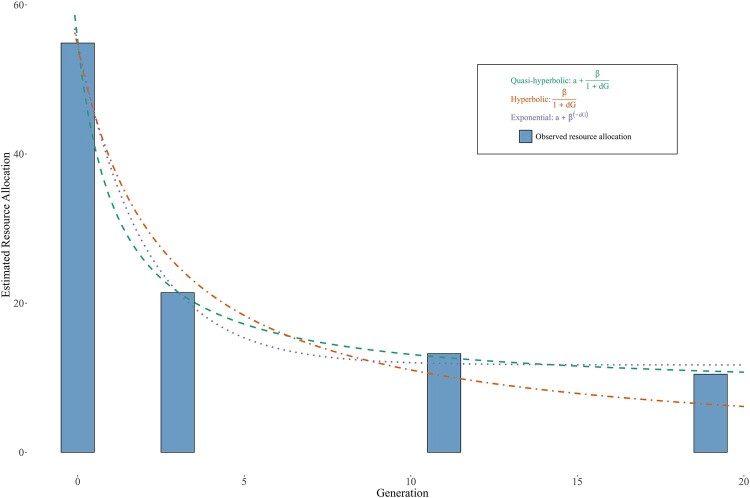
The shape of IGA. The plot depicts the average share of resources devoted to each generation. The graph also shows optimally fitted hyperbolic, quasihyperbolic, and exponential discount functions.

In Fig. 2, we display the average allocation of resources across generations. We also depict the shapes of three discount functions typically used to model decision-making: the hyperbolic, quasihyperbolic, and exponential discount functions, which are fitted to the data so as to minimize prediction errors.^g^ As is clear from Fig. 2, participants devote a disproportionate amount of resources to the present generation, and the share of resources per generation monotonically decreases with temporal (social) distance. The quasihyperbolic discount function appears to best explain the shape of IGA, with an $r^{2}$ of 0.457. We can formally compare the fit of the quasihyperbolic function to the fit of the second-best performing model, namely the exponential function, through a single-factor ANOVA test. The results of this test indicate a statistically significant difference, albeit a marginal one, suggesting that the quasihyperbolic discount function provides a better model for individual resource allocations (degrees of freedom model = 1, degrees of freedom residuals = 12,918, *F*-value = 7.547, *P*-value = 0.006). The estimated parameters of the quasihyperbolic discount function, which indicate present bias and social discounting, are highly statistically significant. This exercise thus provides evidence of both regularity and interpersonal variation in intergenerational altruism.

The experiment did not entail a monetarily incentivized tradeoff, as actual transfers to temporally distant generations would have been difficult to implement (although see Ref. (8) for an innovative approach introducing real stakes in intergenerational decisions). Yet, recent experimental studies have confirmed that indicators of moral universalism are not meaningfully different in incentivized tasks compared with nonincentivized tasks (57, 58). We further validate our operationalization of IGA by correlating distributions in the resource allocation game with a range of participant characteristics. Table S5 shows results that align with theoretical predictions, inter alia, that the share of resources allocated to future generations is higher for participants who are younger, more highly educated, and who support the Swedish Green Party.

### Intergenerational altruism and climate policy preferences

**Fig. 3.**
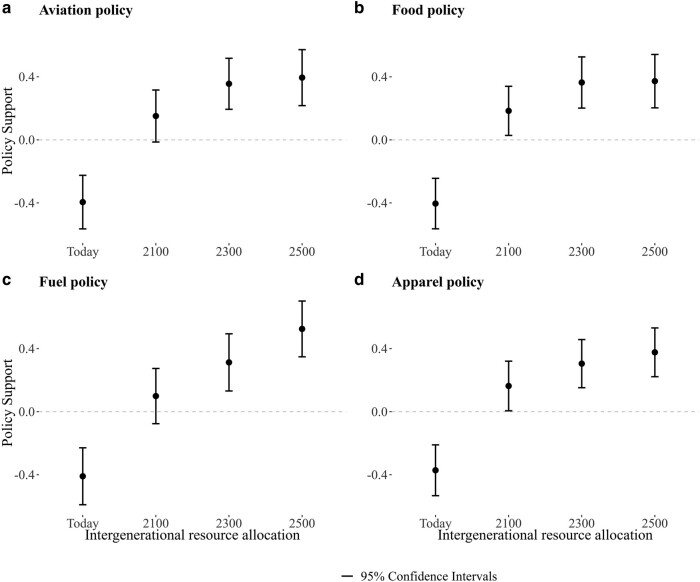
Depicts the correlations between resource allocations across generations and climate policy support. Resource allocations are first residualized on participants' age, income, education, gender, county, and city size. The error bars represent 95% confidence intervals.

Next, we turn to the statistical association between IGA and climate policy preferences. To visualize this relationship, we regress climate policy support for respective policies on resource allocations to each generation—conditional on a range of controls discussed below—and display the estimates in a coefficient plot. The findings are shown in Fig. 3.

As is clear from Fig. 3, there is a positive and monotonic relationship between devoting resources to generations that will live in the distant future and climate policy preferences. The relationships are, reassuringly, almost identical for all four policy scenarios. The fact that devoting more resources to temporally distant generations is associated with stronger support for contemporary climate policies illustrates the long-term moral nature of climate mitigation efforts.

Because we lack exogenous variation in IGA, this analysis cannot establish the causal direction of the relationship. However, by accounting for various factors that potentially could be associated with both CPP and IGA—namely age, income, education, gender, county of residence, and level of urbanicity—we can rule out confounding influences from those variables. Figure 3 depicts the conditional associations. In Table S6, we show that the coefficients are remarkably stable regardless of whether we include controls or not. This exercise demonstrates the robustness of the relationship between IGA and climate policy preferences, thereby also showcasing the independent predictive power of intergenerational altruism.

### The causal effects of the intergenerational dilemma

According to Markowitz and Shariff (10), we may discount the needs of future generations because they are socially distant from us. This conjecture was confirmed by the resource allocations made by participants in our survey (Section 4.1). Moreover, Section 4.2 demonstrated that IGA strongly correlates with CPP. Taken together, if we can reduce the perceived social distance of future generations, support for costly climate policies might correspondingly increase.

**Fig. 4.**
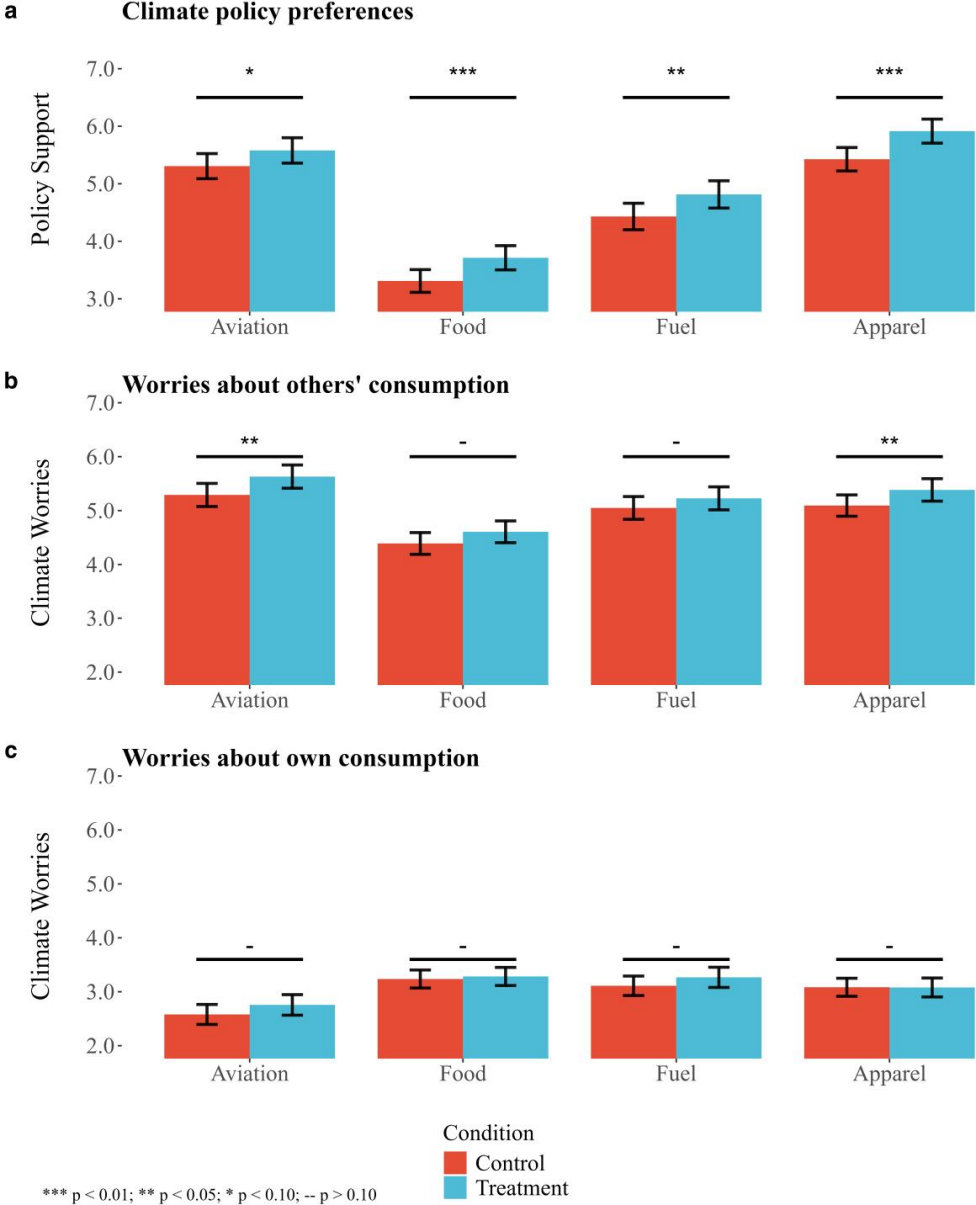
The impact of the intergenerational dilemma on climate policy preferences and worries. Panel (a) shows how exposure to the intergenerational dilemma impacts climate policy support. Panel (b) displays the effect of the intergenerational dilemma on worries about the climatic impact of others’ consumption. Panel (c) shows the impact of the intergenerational dilemma on worries about the climatic impact of own consumption. The error bars represent 95% CIs.

To test this conjecture, we randomly assigned half of the participants to complete the intergenerational dilemma before the climate policy vignettes. We argue that participation in the intergenerational dilemma has the potential to reduce the perceived social distance of future generations as well as to enhance the salience of their needs. The first component of the dilemma constitutes a personalized note informing participants of their projected number of descendants over the next 250 years. The underlying rationale is that, by leveraging the logic of kinship morality and “selective recognition of who is and who is not a kinsman” (59, p. 81), we can reduce the perceived social distance of temporally distant generations. The second component of the dilemma served to increase the salience of the needs of future generations, another feature that might promote the prioritization of distant generations’ welfare. Figure 4a illustrates the impact of participating in the intergenerational dilemma on CPP. Figure 4b and c, on the other hand, displays the effect of the intergenerational dilemma on climatic impact worries related to others’ and own consumption, respectively.

The results in Fig. 4a reveal that participating in the intergenerational dilemma increases CPP across all four policy scenarios. The estimated effect on aviation policy support falls below conventional significance levels, but in all other instances the estimated coefficients are both statistically significant and economically meaningful. In Table S7, we present regression estimates and find that the treatment effect on average policy support is around 0.4 (on a 10-point scale), or about 15% of an SD (column 5). We display the bivariate relationships in Panel A and repeat the analyses in Panel B with controls for gender, age, income, education, county of residence, and city size, the random order in which participants completed the policy vignettes, and the randomized cost treatments (described in Section A2). As should be expected given the randomized nature of the treatment, the findings remain similar both with and without the inclusion of controls. Furthermore, we show that participating in the intergenerational dilemma increased policy support regardless of cost treatment (low, medium, or high markup [Table S8]).

Next, we turn to a candidate mechanism, namely the potential that participation in the intergenerational dilemma increases concerns about the climatic impact of consumption (climatic impact worries) and, as a result, enhances support for climate policies. The results are shown in Fig. 4b and c and replicated in Table S9. Two insights stand out: first, participants report substantially lower levels of climatic impact worries related to their own consumption as compared to others’ consumption; second, the intergenerational dilemma appears to increase climatic impact worries related to others’ consumption of flights and clothing. However, the inconsistent patterns observed, particularly the lack of significant treatment effects on concerns related to food and fuel consumption, complicate the evaluation of the role of climatic impact worries. In the following subsection, we gender-decompose the analysis and find more conclusive patterns.

### Gendered effects of the intergenerational dilemma

**Fig. 5.**
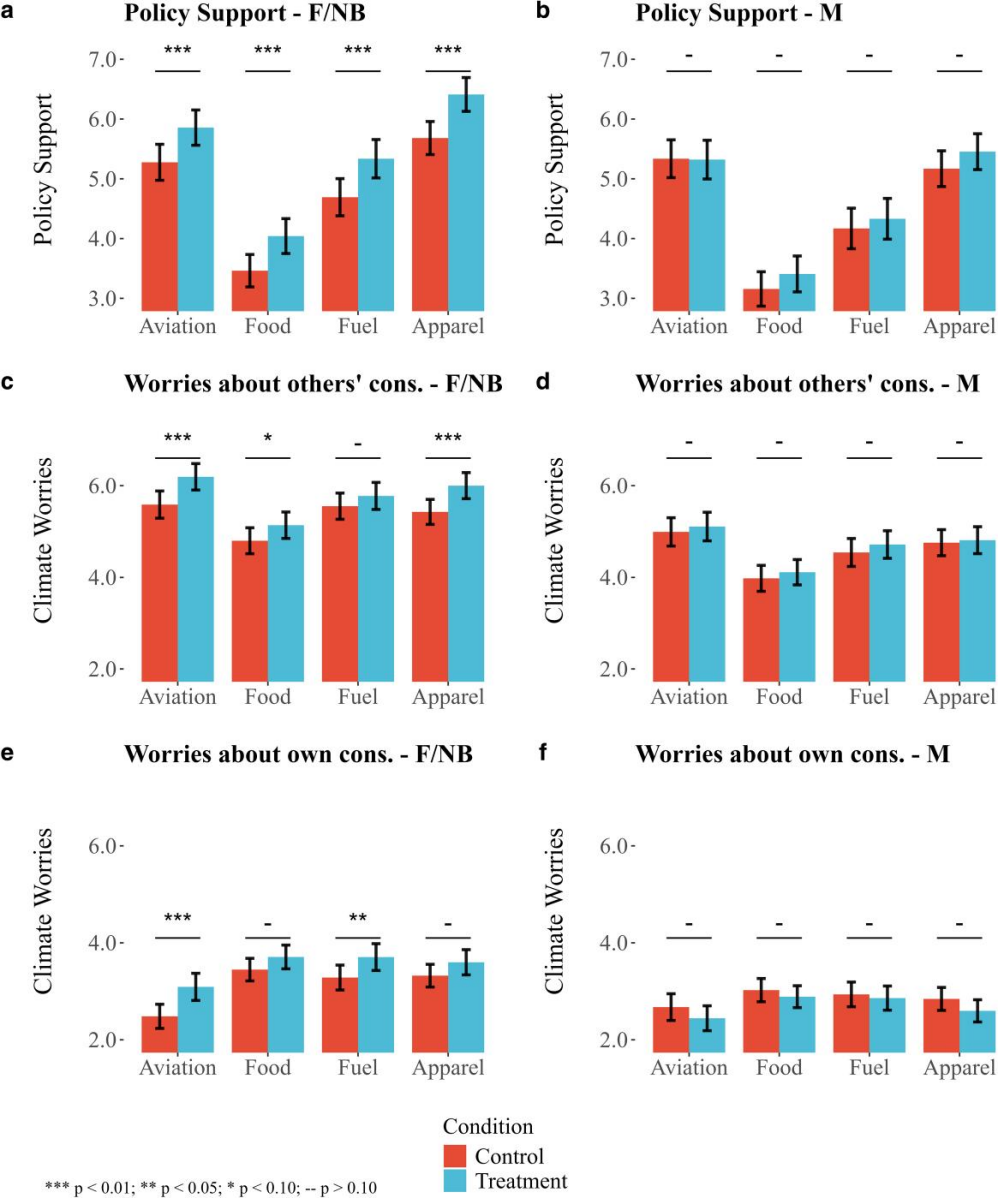
The gendered effect of the intergenerational dilemma. a and b) Exposure to the intergenerational dilemma impacts climate policy support among women and nonbinary but not among men. c and d) Participating in the dilemma enhances climatic impact worries regarding others’ consumption among women and nonbinary but not among men. e and f) Intergenerational dilemma influences climatic impact worries regarding own consumption among women and nonbinary, whereas treated men display lower levels of climatic impact worries compared to nontreated men (estimated below conventional significance levels). The error bars represent 95% CIs.

Building on a rich scholarship on the role of gender norms in shaping attitudes toward climate policies (60–62), we embark on an exploratory investigation of gender-specific effects of the intergenerational dilemma on climate policy preferences. A priori, the role of gender is theoretically ambiguous. Reviewing the literature on gender differences in social preferences, Niederle (63, p. 6), concludes that “while some studies find women to be more altruistic than men, this is not always the case, and differences, when they exist, are often small”. In line with this notion, Table S5 shows that women devote slightly more resources to future generations on average, but this difference is statistically insignificant. At the same time, Croson and Gneezy (64, p. 448), argue that “women are neither more nor less socially oriented, but their social preferences are more malleable”, which might result in gender differences in the response to the intergenerational dilemma. In Fig. 5, we redo the experimental analysis separately for women/nonbinary^h^ (left-hand side) and men (right-hand side).

The results reveal a striking difference in how female/nonbinary participants react to the treatment compared with men. Panels a and b show that while the impact of participating in the intergenerational dilemma on support for each of the four policies is strongly statistically significant for women/nonbinary, men do not exhibit any statistically significant responses (see also Table S10). In other words, the baseline impact of the intergenerational dilemma on policy support is seemingly driven by participants who identify as women or nonbinary.

The same pattern is evident for the impact of the intergenerational dilemma on climate impact worries (Fig. 5c–f). While female/nonbinary participants in the treatment group display higher levels of climatic impact worries compared with female/nonbinary participants in the control group (see also Table S11), the treatment effects for men are all statistically indistinguishable from zero (Table S12). Interestingly, the signs of the coefficients indicate that men report *lower* worries regarding the climatic impact of their own consumption when treated (Table S12, Panel B), which could hint at cognitive dissonance reduction (65). Note, however, that the negative coefficients are estimated below conventional significance levels.

The hypothesis that the intergenerational dilemma enhances support for climate policies among women partly *because* of its impact on climate worries is supported by Table S13. In this table, we present regression analyses that include climatic impact worries as controls and demonstrate that the treatment’s impact on climate policy support is weaker when climate worries are accounted for. In Figs. S4 and S5, we decompose the direct and indirect (mediated) effects of the intergenerational dilemma using the R-package *mediation* (66). Climatic impact worries are shown to significantly mediate the impact of the treatment on support for apparel and aviation policies, whereas the average causal mediated effects (ACME) fall just below conventional significance levels for fuel and food policies. The exercise is solely undertaken for women and nonbinary participants since the analysis revealed no statistically significant reduced-form effect of the intergenerational dilemma on men’s policy preferences.

Finally, we formally test gender-specific responses to participating in the intergenerational dilemma through interaction specifications. In column 1 of Table S14, we demonstrate that the interaction term between the intergenerational dilemma and women/nonbinary is positively associated with policy support but falls just below conventional significance thresholds (borderline significant at 10%). In column 2, we find a positive and statistically significant interaction between gender and the intergenerational dilemma when the outcome is “worries about climatic consequences of own consumption”. Lastly, in column 3, the interaction between treatment and gender is shown to be positively but insignificantly associated with worries regarding others’ consumption. In sum, the gender decomposition reveals intriguing but inconclusive patterns. While female/nonbinary participants appear to be more affected by participating in the intergenerational dilemma than men, the present study lacks sufficient power to either statistically corroborate or reject this tendency. A gender-focused analysis of (the malleability of) IGA constitutes a promising avenue for future research.

## Discussion

In this study, we presented a new approach to empirically measuring IGA and demonstrated the importance of this moral dimension in explaining interpersonal variation in CPP. We leveraged exogenous variation resulting from random assignment to an experimental task, labeled the *intergenerational dilemma*, to identify a causal link between intergenerational moral deliberations and climate policy support. Finally, we provided suggestive evidence of both a mediator (climatic impact worries) and a moderator (gender).

As was argued by Markowitz and Shariff (10), human morality is not wired to account for the needs of generations that will inhabit the planet in a temporally distant future. The shape of IGA revealed in the present study exhibits both present bias and social discounting, which increases with temporal distance. Yet, the fact that participation in the intergenerational dilemma increased participants’ willingness to support (personally costly) climate policies indicates that our moral concerns about the welfare of future others can change.

The present study entails important limitations. First, the abstract nature of the intergenerational resource allocation game implies substantial noise in our operationalization of IGA. Future studies could complement the present experimental approach by including, e.g. concrete examples and monetary incentives. Second, because of the limited sample size, the experimental design comprises only two treatment arms and thus does not allow for the decomposition of the relative influence of the two treatment components—(i) information about the participant-specific projected number of descendants and (ii) the intergenerational resource allocation game. Although this feature of the design does not reduce confidence in the reduced form impact of the intergenerational dilemma, it muddles the interpretation of this effect. More work is needed to dissect the causal pathways between the intergenerational dilemma and climate policy preferences and the potential role that legacy concerns (28) and generativity (43) might play. Third, the heterogeneity analysis based on gender is exploratory, a feature that, in principle, should reduce our confidence in these results. However, the fact that gendered effects are evident across four separate policy scenarios is indicative of heterogeneity, underscoring the importance of more targeted work on gender differences in moral behavior (64).

The methodological and empirical contributions presented in this paper open new avenues for both scholars and policymakers. Advocates for more ambitious climate policies should consider not only the deficiencies but also the opportunities of human morality in dealing with climate change. Given that merely participating in an intergenerational dilemma can increase climate policy support, a better understanding of the moral foundations of climate attitudes could pave the way for more effective climate policy communication.

## Supplementary Material

pgae105_Supplementary_Data

## Data Availability

The data and code necessary to reproduce the findings are deposited at Zenodo https://doi.org/10.5281/zenodo.10635718.

## Notes

a In response to a predominantly myopic research agenda, recent work on the philosophy of longtermism has aimed at shifting focus toward the compound effects of climate mitigation efforts on the welfare of future generations (13, 14).

b The preference has also been labeled intergenerational beneficence (23, 49).

c The equation describes a utility function that depends on the consumption of the present generation, $c_{0}$, and the discounted stream of all future generations’ consumption, $\sum_{t}^{\infty }c_{t}$.

d Later in the paper, we demonstrate that IGA indeed takes a quasihyperbolic functional form.

e Calculated through the following formula, which assumes that each descendant gets two kids: $\sum_{i=1}^{10}(2^{i})$ × Num(Planned)Children.

f In order to increase the perceived consequentiality of the policy scenarios, we informed participants that the results would be reported in scientific as well as nonscientific magazines. According to Carson and Groves (54), the quality of responses hinge on whether participants perceive their answers as important or not.

g We use the nlsLM function in R provided by Elzhov et al. (56) to estimate the parameters that minimize the differences between predicted and actual resource allocations.

h We analyze women and nonbinary as a single group since the low number of nonbinary participants (n=4) prevents a separate analysis for this sample. The results are unchanged if women are analyzed as a separate subgroup.
