# Supplementary material for: Intergenerational altruism and climate policy preferences
Source: PNAS Nexus. 2024 Mar 13;3(4):pgae105. doi: 10.1093/pnasnexus/pgae105 (PMC10986749; doi:10.1093/pnasnexus/pgae105)
Supplement: pgae105_Supplementary_Data [file pgae105_supplementary_data.pdf]

ONLINE APPENDIX

Intergenerational Altruism and Climate Policy Preferences

Table of Contents

A Survey **2**

A.1 Intergenerational dilemma . . . 2

A.2 Climate policy preferences . . . 3

A.3 Climatic impact worries . . . 5

A.4 Data collection . . . 5

B Data **6**

C Additional results **10**

A Survey

A.1 Intergenerational dilemma

The survey included a randomized element in which half the participants were exposed to (i) information about their projected number of descendants over the next 250 years, and (ii) an adapted resource allocation game which asked participants to distribute fictional resources across generations. The combination of (i) and (ii) constitutes the intergenerational dilemma.

In the first component of the intergenerational dilemma, participants were asked to state their current and planned number of children. For instance, if a given participant currently had two children but wanted one more, they were instructed to answer three. Assuming a fertility rate of two for all future generations and an average length of generations of 25 years, we calculate the projected number of descendants in the next 250 years using the following formula:

$$ProjectedDescendants = \sum_{i=1}^{10}(2^i) \times \text{Num(Planned)Children} \tag{1}$$

Next, we informed participants about this projection through the following brief statement:

> **Projected descendants**
> If your (potential) descendants have two children on average, you will have about {ProjectedDescendants} descendants in the next 250 years.

Given that we assume (approximate) replacement level fertility, the number of projected descendants is a linear function of the number of planned children: if a participant planned 1 child, they were informed that they would have *1016* descendants in the next 250 years, and if they answered 3 children, they were informed that they would have *3048* descendants. The information served to reduce the perceived social distance between exposed participants and future generations by emphasizing their shared affiliation.

In the second component of the dilemma, participants were asked to complete a resource allocation game in which they should allocate resources between humans alive today as well as those alive in the years 2100, 2300, and 2500. The resource allocation game served to highlight the needs of future generations and to underscore the trade-off inherent in prioritizing the needs of the present generation. The instructions for the intergenerational resource allocation game, along with an exemplified distribution, are shown in Figure A1.

The questions, information, and resource allocation game, which constitute the intergenerational dilemma, were intended to make our affinity with and the needs of future generations salient. Approximately half of the participants were exposed to it before a set of vignettes probing support for different climate policies, whereas half completed it afterward. This (exogenous) between-subject variation in the salience of intergenerational concerns underpins our identification strategy.

Figure S1: Example of an intergenerational resource allocation with present bias

Now imagine that you have 100 "assets" to distribute between different generations. More assets provide the opportunity for better prosperity. How would you choose to distribute these assets between the following generations?
("People alive today" includes yourself, your loved ones, and everyone else alive today)

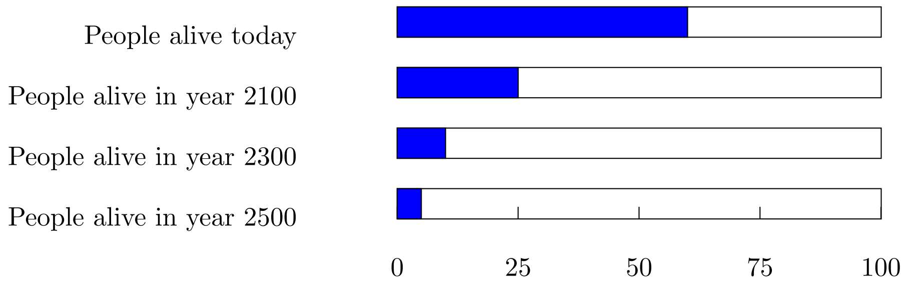

A.2 Climate policy preferences

The vignettes described distinct policy proposals aimed at mitigating consumption-induced greenhouse gas emissions. Specifically, the survey encompassed policies directed at tackling the climatic externalities of aviation, food, car fuel, and apparel consumption. Table S1 outlines the exact wording of each policy proposal. The order in which participants were exposed to the policy scenarios was randomized.

To provide for a moral/material trade-off, the vignettes stated that actual implementation of the policies would result in higher consumption costs for the respective goods. We randomized the cost increases (markups) so that each policy came with a low, medium, or high markup. Personal cost increases were then calculated as the (randomized) markup factors multiplied by participants' private consumption of respective goods, which we elicited through budget questions at the beginning of the survey. The randomized cost treatments reduced climate policy support as expected but are not further analyzed in this paper. Because the randomization of cost treatments was orthogonal to the intergenerational dilemma, they cannot threaten the internal validity of our main findings. To demonstrate the invariance of our findings, we control for the cost treatments in the main specification and show that the intergenerational dilemma impacts climate policy preferences in the different cost scenarios (Online Appendix Table S8). After the participants read each policy proposal, we gauged their support through the following question:

Climate policy support
How likely are you to support the policy proposal regarding [removed aviation subsidies; a climate tax on food; higher share of biofuels; making apparel companies pay for the costs of their emissions]? [0 (extremely unlikely) - 10 (extremely likely)]

The continuous scale, ranging from 0-10, ensures meaningful between-subject variation in policy support.

Table S1: Climate Policy Scenarios and Cost Conditions

**Aviation Policy**	**Food Policy**
Emissions from Swedes' air travel amounted to 10 million tonnes of carbon dioxide equivalents in 2019, just over a tenth of Swedes' consumption emissions of greenhouse gasses. Today, the aviation industry is heavily subsidized and pays no fuel, energy or carbon taxes, is exempt from VAT, and operates at publicly subsidized airports.<br>One day you read about a bill that aims to remove these subsidies to reduce the impact of Swedish aviation on the climate. According to experts, the proposal could mean that the cost of air tickets increases by an average of [*20/40/80*] percent. Your personal annual cost for air travel would thus increase by approximately XXX SEK.	Emissions from Swedes' food consumption amounted to 15 million tonnes of carbon dioxide equivalents in 2019, just over a seventh of Swedes' consumption emissions of greenhouse gasses. Emissions occur in all stages: fertilizer production, animal husbandry, cultivation, harvest, food packaging, and transportation.<br>One day you read about a climate tax on food that aims to reduce the impact of food consumption in Sweden on the climate. According to experts, the proposal could mean that the cost of food increases by approximately [*4/8/16*] percent, thus increasing your personal annual cost for food by approximately XXX SEK.
**Fuel Policy**	**Apparel Policy**
Emissions from Swedes' car transport were almost 10 million tonnes of carbon dioxide equivalents in 2020, just over a tenth of Swedes' consumption emissions of greenhouse gasses. The reduction obligation means that the fuel that is used by cars in Sweden is mixed with biofuels, which reduces its climate impact.<br>One day you read about a bill that aims to reduce emissions from car traffic by further increasing the share of biofuels in fuel. According to experts, the proposal could mean that the cost of fuel increases by an average of [*3/6/12*] percent, thus increasing your personal annual cost for car transport by approximately XXX SEK.	Emissions from Swedes' apparel consumption were almost 4.2 million tonnes of carbon dioxide equivalents in 2020, almost one twentieth of Swedes' consumption emissions of greenhouse gasses. Emissions occur in all stages: material, production, and transportation.<br>One day you read about a bill that aims to reduce emissions from textile consumption through a new framework that requires apparel companies to compensate for emissions. The proposal would mean a cost increase of [*10/20/40*] percent on the new apparel and shoes you buy. It would increase the cost of your apparel consumption by approximately XXX SEK per year.

Note: Table S1 displays the four climate policy scenarios. The order in which participants were exposed to the different vignettes was randomized. The increased consumption cost (XXX Swedish krona (SEK)) was calculated by multiplying participants' self-reported consumption costs of respective goods with the markup factor (low, medium, or high cost scenario).

A.3 Climatic impact worries

After participants stated their policy support (we chose this ordering of questions to ensure clean variation in the key outcome variable), we gauged their worries about the climatic consequences of private consumption of respective goods. Specifically, we asked:

Climatic impact worries
How worried are you about the climatic impact of (1) yours and (2) others' [air travel; food consumption; car transportation; apparel consumption]? [0 (Not at all worried) - 10 (extremely worried)]

Participants provide separate responses for worries about the climatic consequences of their own and others consumption.

A.4 Data collection

We sampled 1615 Swedish adults from an online (nationally representative) pool. Participants were selected at random by means of a stratified sample process (stratified by age, gender, and county). As a result, participant demographics such as age, gender, and place of residence closely resemble those of the broader Swedish population (see the Online Appendix Table S2). Participants received a small payment for completing the online survey.

Table S2: Sample representativity

Panel A: Age		
Age group	Population	Survey
18-25	11	10
26-35	18	21
36-45	16	12
46-55	16	16
56-65	15	15
66+	24	25
**Panel B: Gender**		
Reported gender[a]	Population	Survey
Male	50	51
Female	50	49

[a]This table reports official gender statistics, which may not always reflect self-identified gender.

B Data

Table S3: Balance tests

	Control	s.d.	Treatment	s.d.	diff.	p-val.
	(1)	(2)	(3)	(4)	(5)	(6)
Male (dum.)	0.499	(0.500)	0.519	(0.500)	0.019	(0.436)
Age (cont.)	49.596	(18.226)	48.132	(18.281)	-1.464	(0.108)
University (dum.)	0.275	(0.447)	0.226	(0.418)	-0.050**	(0.021)
Income (cont.)	26,053.352	(13,215.232)	25,650.969	(13,113.928)	-402.382	(0.560)
City Size (cont.)	2.886	(1.071)	2.752	(1.055)	-0.134**	(0.011)
Planned Kids (cont.)	1.759	(1.330)	1.799	(1.168)	0.040	(0.526)
Flight Budget (cont.)	1,282.186	(1,011.040)	1,387.578	(1,171.289)	105.392*	(0.053)
Food Budget (cont.)	4,212.974	(8,403.362)	3,562.657	(8,006.682)	-650.318	(0.111)
Fuel Budget (cont.)	1,278.091	(1,340.059)	1,312.594	(1,305.178)	34.503	(0.600)
Clothes Budget (cont.)	522.399	(698.370)	516.917	(725.353)	-5.482	(0.877)
Green Party (dum.)	0.043	(0.203)	0.045	(0.208)	0.002	(0.824)
Sweden Democrats (dum.)	0.185	(0.388)	0.173	(0.378)	-0.012	(0.533)
Social Democrats (dum.)	0.268	(0.443)	0.296	(0.457)	0.028	(0.217)
Stockholm (dum.)	0.224	(0.417)	0.226	(0.418)	0.002	(0.940)
Observations	817		798		1,615	

Note: Table S3 displays balance tests. Columns 1 and 3 show the mean value of each variable in the different conditions. In Columns 2 and 4, the corresponding standard deviations are shown. Column 5 displays the estimated differences and column 6 the corresponding p-values based on robust standard errors.

Table S4: Variable descriptions

Label	Survey item
**Policy support**	
	How likely are you to support the policy proposal regarding...
Aviation policy support	... removed aviation subsidies [0 (extremely unlikely) - 10 (extremely likely)]
Food policy support	... a climate tax on food [0 (extremely unlikely) - 10 (extremely likely)]
Fuel policy support	... higher share of biofuels [0 (extremely unlikely) - 10 (extremely likely)]
Apparel policy support	... making apparel companies pay for the costs of their emissions [0 (extremely unlikely) - 10 (extremely likely)] ?
**Climatic impact worries**	
	How worried are you about the climatic impact of (1) yours and (2) others'...
Aviation worries	... air travel [0 (Not at all worried) - 10 (extremely worried)]
Food worries	... food consumption [0 (Not at all worried) - 10 (extremely worried)]
Fuel worries	... car transportation [0 (Not at all worried) - 10 (extremely worried)]
Apparel worries	... apparel consumption [0 (Not at all worried) - 10 (extremely worried)]
**Budget questions**	
Aviation budget	In the last 12 months, approximately how much did you spend in total on air travel? (For charters, count with flight tickets as constituting 50% of the total cost. Include costs for all plane tickets that you paid personally, for example also for any children) [multiple alternatives]
Food budget	In the last 12 months, approximately how much did you spend on food shopping, dining out and take-away on average per week? (include all food expenses) [multiple alternatives]
Fuel budget	In the last 12 months, approximately how much did you spend on fuel on average per month? (include costs for fuel for own and other people's car(s))[multiple alternatives]
Apparel budget	In the last 12 months, approximately how much did you spend on new clothes per month on average? [multiple alternatives]
**Control variables**	
Age	How old are you? [Open answer]
Gender identity	What gender do you identify with? [Woman, Man, Non-binary, Other, Unsure, Do not want to answer]
Income	What is your average monthly disposable income? Include all types of incomes *after* tax. [Multiple categories]
Education	What is your highest level of education achieved so far? [Multiple categories]
County	Which county do you live in? [Multiple categories]
City size	How many inhabitants live in the city where you currently reside? [Multiple categories]

Note: In Table S4, the survey variables that were employed in the empirical study are described.

Figure S2: Intergenerational altruism

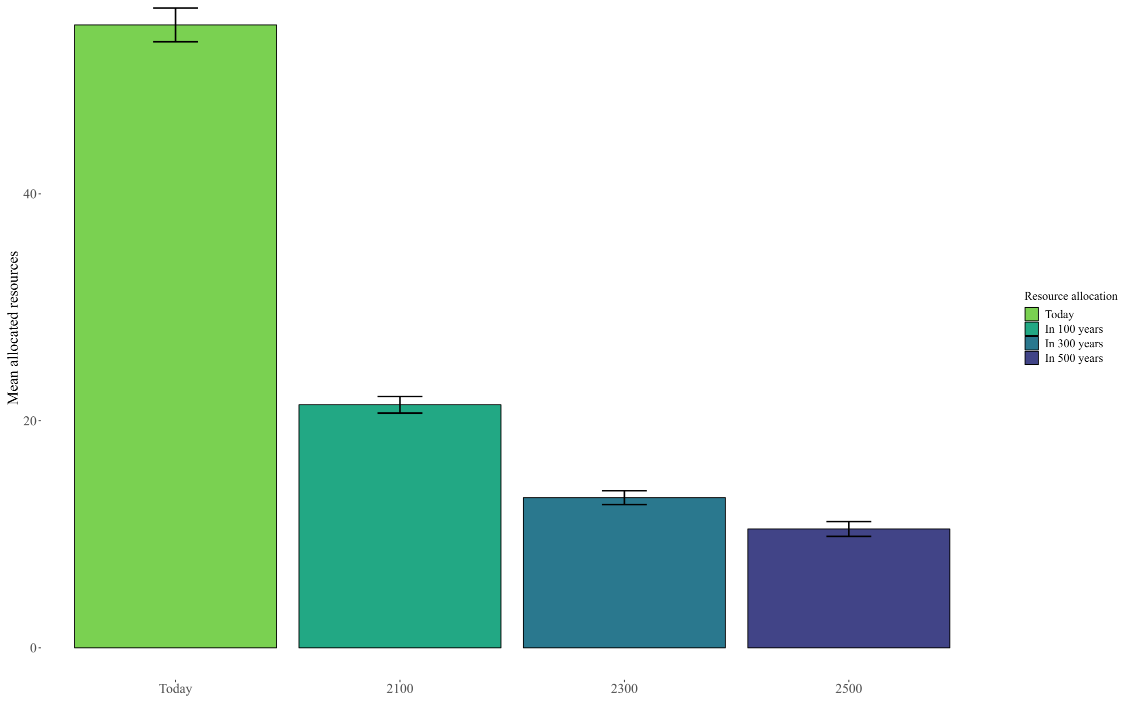

Note: In Figure S2 we display the average distribution of resources between generations. The error bars represent 95% confidence intervals.

Figure S3: Climate policy preferences

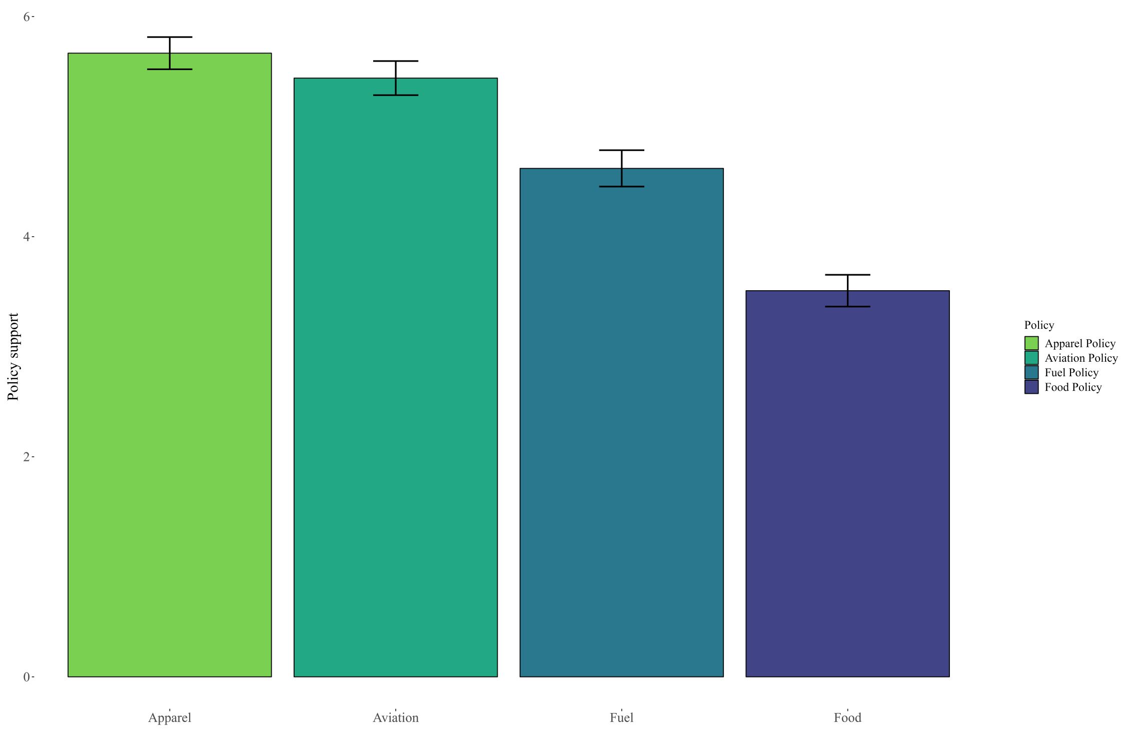

Note: In Figure S3 we display the average support for each climate policy. The error bars represent 95% confidence intervals.

C Additional results

Table S5: The association between participants' traits and their intergenerational altruism

**Panel A.** Dep. Var.:	Resources today			
	(1)	(2)	(3)	(4)
Male	2.208			
	(1.499)			
Age	0.255***			
	(0.0408)			
University (dummy)		-5.860***		
		(1.866)		
Income (continuous)		0.000124**		
		(0.0000623)		
Urban		-4.718***		
		(1.673)		
Flight budget			0.0000752	
			(0.000102)	
Food budget			0.000492	
			(0.000722)	
Fuel budget			0.00145**	
			(0.000599)	
Clothes budget			-0.000594	
			(0.00112)	
Sweden Democrats				5.573***
				(1.949)
Green Party				-8.223**
				(3.216)
Constant	41.27***	54.91***	52.37***	54.25***
	(2.192)	(1.835)	(1.310)	(0.866)
Observations	1613	1453	1615	1615
R-squared	0.0245	0.0141	0.00503	0.00877
Dep. Var. Mean	54.86	55.09	54.89	54.89
Dep. Var. Std.	30.44	30.37	30.44	30.44
**Panel B.** Dep. Var.:	Inverted RAG slope			
	(1)	(2)	(3)	(4)
Male	0.0613			
	(0.0494)			
Age	0.00736***			
	(0.00136)			
University (dummy)		-0.191***		
		(0.0625)		
Income (continuous)		0.00000394*		
		(0.00000205)		
Urban		-0.165***		
		(0.0561)		
Flight budget			0.000000738	
			(0.00000335)	
Food budget			0.0000274	
			(0.0000235)	
Fuel budget			0.0000522***	
			(0.0000192)	
Clothes budget			0.000000641	
			(0.0000359)	
Sweden Democrats				0.214***
				(0.0624)
Green Party				-0.258**
				(0.117)
Constant	-0.392***	0.00255	-0.107**	-0.0270
	(0.0737)	(0.0604)	(0.0430)	(0.0285)
Observations	1613	1453	1615	1615
R-squared	0.0188	0.0145	0.00695	0.0104
Dep. Var. Mean	-0.000842	0.00183	-4.42e-09	-4.42e-09
Dep. Var. Std.	1.000	1.001	1.000	1.000

Robust standard errors in parentheses
* $p < 0.10$, ** $p < 0.05$, *** $p < 0.010$

Note: Table S5 displays OLS regression estimates of the statistical associations between participants' traits and intergenerational altruism. Intergenerational altruism is proxied by resources devoted to today's geeneration in the intergenerational resource allocation game (Panel A) and by the (standardized) inverted slope coefficient of resource distributions across generations (i.e., a measure of how much resource allocation dissipates with temporal distance) (Panel B). Robust standard errors in parentheses.

Table S6: Climate policy support and intergenerational altruism

**Panel A.** Dep. Var.:	Aviation PS	Food PS	Fuel PS	Apparel PS	Avg. PS
	(1)	(2)	(3)	(4)	(5)
Resources today	-0.0160***	-0.0192***	-0.0209***	-0.0181***	-0.0186***
	(0.00292)	(0.00267)	(0.00300)	(0.00271)	(0.00232)
Constant	5.996***	4.134***	5.329***	6.204***	5.416***
	(0.162)	(0.155)	(0.173)	(0.150)	(0.131)
Observations	1615	1615	1615	1615	1615
R-squared	0.0218	0.0375	0.0330	0.0351	0.0453
Dep. Var. Mean	5.441	3.509	4.620	5.667	4.809
Dep. Var. Std.	3.176	2.959	3.387	2.997	2.601
Intergenerational dilemma	Yes	Yes	Yes	Yes	Yes
Controls	No	No	No	No	No
**Panel B.** Dep. Var.:	Aviation PS	Food PS	Fuel PS	Apparel PS	Avg. PS
	(1)	(2)	(3)	(4)	(5)
Resources today	-0.0184***	-0.0213***	-0.0220***	-0.0201***	-0.0207***
	(0.00311)	(0.00292)	(0.00320)	(0.00284)	(0.00249)
Constant	5.043***	3.370***	4.226***	5.808***	4.032***
	(0.742)	(0.730)	(0.895)	(0.693)	(0.611)
Observations	1449	1448	1448	1449	1451
R-squared	0.111	0.111	0.151	0.145	0.144
Dep. Var. Mean	5.464	3.537	4.705	5.676	4.838
Dep. Var. Std.	3.191	2.982	3.412	3.015	2.637
Intergenerational dilemma	Yes	Yes	Yes	Yes	Yes
Controls	Yes	Yes	Yes	Yes	Yes

Robust standard errors in parentheses
* $p < 0.10$, ** $p < 0.05$, *** $p < 0.010$

Note: Table S6 displays OLS regression estimates of the association between intergenerational altruism and climate policy preferences. *International dilemma F.E.* indicates fixed effects for the intergenerational dilemma. *Controls* indicate fixed effects for gender, income category, educational category, county, and city size, as well as a continuous control for age. Robust standard errors in parentheses.

Table S7: Intergenerational dilemma and climate policy support

**Panel A.**					
Dep. Var.:	Aviation PS	Food PS	Fuel PS	Apparel PS	Avg. PS
	(1)	(2)	(3)	(4)	(5)
Intergenerational dilemma	0.273*	0.403***	0.384**	0.489***	0.387***
	(0.158)	(0.147)	(0.168)	(0.149)	(0.129)
Constant	5.306***	3.310***	4.431***	5.426***	4.618***
	(0.111)	(0.101)	(0.117)	(0.104)	(0.0891)
Observations	1615	1615	1615	1615	1615
R-squared	0.00185	0.00465	0.00321	0.00665	0.00554
Dep. Var. Mean	5.441	3.509	4.620	5.667	4.809
Dep. Var. Std.	3.176	2.959	3.387	2.997	2.601
Cost Treatment & Order F.E.	No	No	No	No	No
Controls	No	No	No	No	No
**Panel B.**					
Dep. Var.:	Aviation PS	Food PS	Fuel PS	Apparel PS	Avg. PS
	(1)	(2)	(3)	(4)	(5)
Intergenerational dilemma	0.305*	0.528***	0.624***	0.699***	0.525***
	(0.166)	(0.157)	(0.173)	(0.154)	(0.135)
Constant	4.559***	2.702***	3.615***	5.263***	3.456***
	(0.759)	(0.736)	(0.907)	(0.720)	(0.632)
Observations	1449	1448	1448	1449	1451
R-squared	0.0876	0.0751	0.121	0.114	0.0997
Dep. Var. Mean	5.464	3.537	4.705	5.676	4.838
Dep. Var. Std.	3.191	2.982	3.412	3.015	2.637
Cost Treatment & Order F.E.	Yes	Yes	Yes	Yes	No
Controls	Yes	Yes	Yes	Yes	Yes

Robust standard errors in parentheses
* $p < 0.10$, ** $p < 0.05$, *** $p < 0.010$

Note: Table S7 displays OLS regression estimates of the impact of the intergenerational dilemma on climate policy preferences. Each column displays the effect on a different policy. In column 5, we display the effect of the intergenerational dilemma on the average of the four policy preferences (Aviation, Food, Fuel, and Apparel policy support). *Cost Treatment & Order F.E.* indicate fixed effects for cost treatments and the order at which participants completed the policy vignettes. *Controls* indicate fixed effects for gender, income category, educational category, county, and city size, as well as a continuous control for age. Robust standard errors in parentheses.

Table S8: The impact of the intergenerational dilemma separately for each cost treatment

Cost treatment:	Low	Medium	High
Dep. Var.:	Avg. PS	Avg. PS	Avg. PS
	(1)	(2)	(3)
Intergenerational dilemma	0.443***	0.293*	0.333**
	(0.169)	(0.164)	(0.164)
Constant	4.901***	4.642***	4.345***
	(0.120)	(0.117)	(0.111)
Observations	1293	1305	1284
R-squared	0.00529	0.00245	0.00323
Dep. Var. Mean	5.123	4.790	4.507
Dep. Var. Std.	3.048	2.965	2.933

Robust standard errors in parentheses
* $p < 0.10$, ** $p < 0.05$, *** $p < 0.010$

Note: Table S8 displays OLS regression estimates of the intergenerational dilemma on the average policy support, separately for each cost treatment (low markup; medium markup; high markup). Robust standard errors in parentheses.

Table S9: Intergenerational dilemma and climate impact worries

**Panel A.** Dep. Var.:	Worry Aviation (Others)	Worry Food (Others)	Worry Fuel (Others)	Worry Apparel (Others)
	(1)	(2)	(3)	(4)
Intergenerational dilemma	0.360**	0.235	0.200	0.307**
	(0.154)	(0.145)	(0.153)	(0.147)
Constant	5.166***	4.504***	4.870***	5.196***
	(0.201)	(0.190)	(0.198)	(0.190)
Observations	1613	1612	1612	1613
R-squared	0.0227	0.00401	0.00273	0.00443
Dep. Var. Mean	5.465	4.506	5.146	5.243
Dep. Var. Std.	3.113	2.918	3.067	2.937
Cost Treatment & Order F.E.	Yes	Yes	Yes	Yes
**Panel B.** Dep. Var.:	Worry Aviation (Own)	Worry Food (Own)	Worry Fuel (Own)	Worry Apparel (Own)
	(1)	(2)	(3)	(4)
Intergenerational dilemma	0.185	0.0558	0.162	0.0141
	(0.136)	(0.120)	(0.133)	(0.124)
Constant	2.596***	3.613***	3.251***	3.317***
	(0.182)	(0.163)	(0.168)	(0.159)
Observations	1613	1612	1612	1613
R-squared	0.00388	0.0168	0.00575	0.00462
Dep. Var. Mean	2.670	3.266	3.194	3.085
Dep. Var. Std.	2.719	2.426	2.664	2.478
Cost Treatment & Order F.E.	Yes	Yes	Yes	Yes

Robust standard errors in parentheses
* $p < 0.10$, ** $p < 0.05$, *** $p < 0.010$

Note: Table S9 displays OLS regression estimates of the effects of the intergenerational dilemma on climate impact worries. Panel A shows the estimated effects on worries about the consumption of others, whereas Panel B shows estimates of the effects on worries about own consumption. Robust standard errors in parentheses.

Table S10: Intergenerational dilemma and climate policy support by gender

**Panel A: women/non-binary**					
Dep. Var.:	Aviation PS	Food PS	Fuel PS	Apparel PS	Avg. PS
	(1)	(2)	(3)	(4)	(5)
Intergenerational dilemma	0.637***	0.612***	0.669***	0.775***	0.633***
	(0.214)	(0.203)	(0.228)	(0.199)	(0.173)
Constant	5.485***	3.759***	5.125***	6.248***	4.778***
	(0.271)	(0.264)	(0.299)	(0.258)	(0.119)
Observations	792	791	791	791	793
R-squared	0.0296	0.0168	0.0181	0.0412	0.0168
Dep. Var. Mean	5.564	3.752	5.016	6.051	5.085
Dep. Var. Std.	3.032	2.860	3.208	2.842	2.446
Cost Treatment & Order F.E.	Yes	Yes	Yes	Yes	No
**Panel B: men**					
Dep. Var.:	Aviation PS	Food PS	Fuel PS	Apparel PS	Avg. PS
	(1)	(2)	(3)	(4)	(5)
Intergenerational dilemma	-0.0185	0.226	0.203	0.268	0.171
	(0.231)	(0.211)	(0.247)	(0.214)	(0.190)
Constant	5.475***	3.363***	4.664***	6.121***	4.458***
	(0.312)	(0.288)	(0.326)	(0.263)	(0.132)
Observations	821	821	821	822	822
R-squared	0.0152	0.0247	0.00724	0.0304	0.000990
Dep. Var. Mean	5.335	3.287	4.256	5.313	4.544
Dep. Var. Std.	3.299	3.034	3.508	3.090	2.717
Cost Treatment & Order F.E.	Yes	Yes	Yes	Yes	No

Robust standard errors in parentheses
* $p < 0.10$, ** $p < 0.05$, *** $p < 0.010$

Note: Table S10 displays OLS regression estimates of the impact of the intergenerational dilemma on climate policy support, separately for women/non-binary (Panel A) and Men (Panel B). Robust standard errors in parentheses.

Table S11: Intergenerational dilemma and climate impact worries: women and non-binary

**Panel A.**				
Dep. Var.:	Worry Aviation (Others)	Worry Food (Others)	Worry Fuel (Others)	Worry Apparel (Others)
	(1)	(2)	(3)	(4)
Intergenerational dilemma	0.622***	0.374*	0.282	0.609***
	(0.210)	(0.205)	(0.209)	(0.201)
Constant	5.301***	5.064***	4.996***	5.446***
	(0.269)	(0.258)	(0.266)	(0.275)
Observations	792	791	791	791
R-squared	0.0314	0.0119	0.0104	0.0159
Dep. Var. Mean	5.888	4.976	5.675	5.719
Dep. Var. Std.	2.974	2.887	2.927	2.820
Cost Treatment & Order F.E.	Yes	Yes	Yes	Yes
**Panel B.**				
Dep. Var.:	Worry Aviation (Own)	Worry Food (Own)	Worry Fuel (Own)	Worry Apparel (Own)
	(1)	(2)	(3)	(4)
Intergenerational dilemma	0.628***	0.285*	0.419**	0.305*
	(0.192)	(0.170)	(0.194)	(0.179)
Constant	2.315***	3.895***	3.406***	3.451***
	(0.244)	(0.225)	(0.242)	(0.230)
Observations	792	791	791	791
R-squared	0.0186	0.0304	0.0111	0.00616
Dep. Var. Mean	2.782	3.583	3.497	3.465
Dep. Var. Std.	2.690	2.417	2.704	2.499
Cost Treatment & Order F.E.	Yes	Yes	Yes	Yes

Robust standard errors in parentheses
* $p < 0.10$, ** $p < 0.05$, *** $p < 0.010$

Note: Table S11 displays OLS regression estimates of the impact of the intergenerational dilemma on climatic impact worries among women/non-binary. In Panel A, we show the effects on worries about the climatic impacts of others' consumption. In Panel B, we present the effects on worries about the climatic impacts of own consumption. Robust standard errors in parentheses.

Table S12: Intergenerational dilemma and climate impact worries: men

**Panel A.**				
Dep. Var.:	Worry Aviation (Others)	Worry Food (Others)	Worry Fuel (Others)	Worry Apparel (Others)
	(1)	(2)	(3)	(4)
Intergenerational dilemma	0.119	0.134	0.205	0.0542
	(0.223)	(0.202)	(0.219)	(0.210)
Constant	5.022***	4.008***	4.665***	4.973***
	(0.297)	(0.269)	(0.289)	(0.262)
Observations	821	821	821	822
R-squared	0.0235	0.00204	0.00499	0.00350
Dep. Var. Mean	5.057	4.052	4.637	4.785
Dep. Var. Std.	3.190	2.877	3.113	2.976
Cost Treatment & Order F.E.	Yes	Yes	Yes	Yes
**Panel B.**				
Dep. Var.:	Worry Aviation (Own)	Worry Food (Own)	Worry Fuel (Own)	Worry Apparel (Own)
	(1)	(2)	(3)	(4)
Intergenerational dilemma	-0.242	-0.143	-0.0553	-0.236
	(0.193)	(0.168)	(0.183)	(0.168)
Constant	2.903***	3.376***	3.036***	3.202***
	(0.269)	(0.231)	(0.232)	(0.218)
Observations	821	821	821	822
R-squared	0.00700	0.0109	0.00492	0.0156
Dep. Var. Mean	2.562	2.960	2.901	2.719
Dep. Var. Std.	2.744	2.398	2.593	2.403
Cost Treatment & Order F.E.	Yes	Yes	Yes	Yes

Robust standard errors in parentheses
* $p < 0.10$, ** $p < 0.05$, *** $p < 0.010$

Note: Table S12 displays OLS regression estimates of the impact of the intergenerational dilemma on climatic impact worries among men. In Panel A, we show the effects on worries about the climatic impacts of others' consumption. In Panel B, we present the effects on worries about the climatic impacts of own consumption. Robust standard errors in parentheses.

Table S13: The impact of the intergenerational dilemma on policy support is weaker when accounting for climate worries (Women/Non-binary)

Sample:	Women/NB	Women/NB	Women/NB	Women/NB
Dep. Var.:	Aviation PS	Food PS	Fuel PS	Apparel PS
	(1)	(2)	(3)	(4)
Intergenerational dilemma	0.248	0.384**	0.481***	0.409**
	(0.175)	(0.166)	(0.183)	(0.161)
Aviation worries (own)	0.00869			
	(0.0345)			
Aviation worries (others)	0.617***			
	(0.0321)			
Food worries (own)		0.207***		
		(0.0581)		
Food worries (others)		0.452***		
		(0.0461)		
Fuel worries (own)			-0.00145	
			(0.0432)	
Fuel worries (others)			0.667***	
			(0.0377)	
Apparel worries (own)				0.117***
				(0.0354)
Apparel worries (others)				0.543***
				(0.0366)
Constant	2.194***	0.666**	1.798***	2.888***
	(0.277)	(0.262)	(0.313)	(0.291)
Observations	792	791	791	791
R-squared	0.388	0.355	0.384	0.393
Dep. Var. Mean	5.564	3.752	5.016	6.051
Dep. Var. Std.	3.032	2.860	3.208	2.842

Robust standard errors in parentheses
* $p < 0.10$, ** $p < 0.05$, *** $p < 0.010$

Note: Table S13 displays OLS regression estimates of the impact of the intergenerational dilemma among women/non-binary when climatic impact worries are accounted for. Robust standard errors in parentheses.

Table S14: The interaction between gender and exposure to the intergenerational dilemma

Dep. Var.:	Avg. Policy Support	Avg. Worries (own)	Avg. Worries (others)
	(1)	(2)	(3)
Intergenerational dilemma	0.171	-0.172	0.119
	(0.190)	(0.148)	(0.189)
Female/Non-binary	0.320^{*}	0.265^{*}	0.773^{***}
	(0.178)	(0.145)	(0.183)
Intergenerational dilemma	0.462^{*}	0.564^{***}	0.316
× Female/Non-binary	(0.256)	(0.210)	(0.260)
Constant	4.458^{***}	2.869^{***}	4.569^{***}
	(0.132)	(0.105)	(0.133)
Observations	1615	1615	1615
R-squared	0.0186	0.0212	0.0337
Dep. Var. Mean	4.809	3.049	5.082
Dep. Var. Std.	2.601	2.127	2.661

Robust standard errors in parentheses
* $p < 0.10$, ** $p < 0.05$, *** $p < 0.010$

Note: Table S14 displays OLS regression estimates of how exposure to the intergenerational dilemma influenced average policy support (column 1), average climatic impact worries regarding own consumption (column 2), and average climatic impact worries regarding others' consumption (column 3). Robust standard errors in parentheses.

Figure S4: Mediation by climatic impact worries for women/non-binary: mediation model

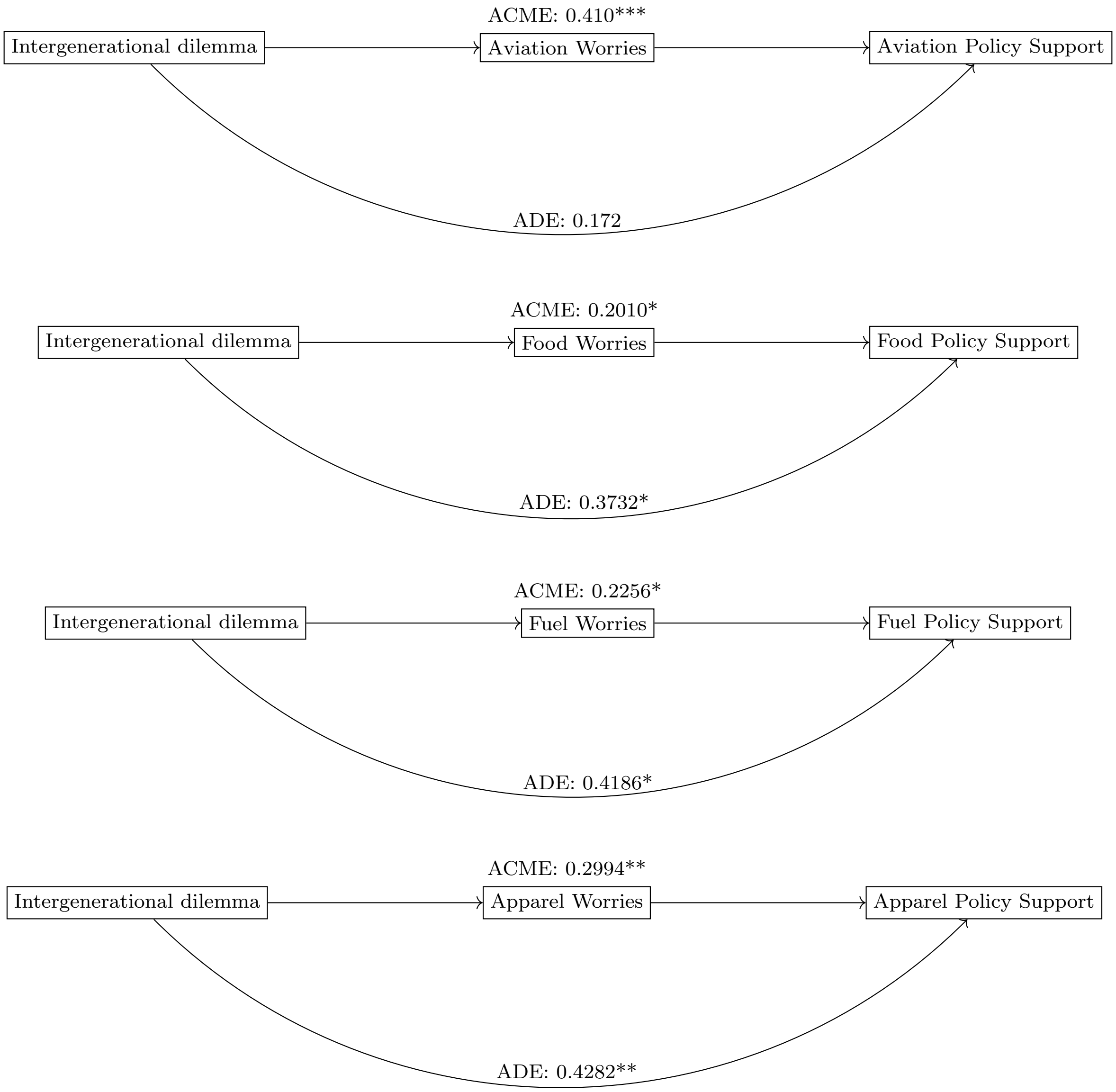

Note: The figure displays the direct (ADE) and mediated (ACME) effects of the intergenerational dilemma on policy support, separately for each policy. The mediating variable is climatic impact worries, which is defined as the average worries regarding both own and others' consumption of respective goods. The sample comprises 793 women and non-binary participants. *** p¡0.01; ** p¡0.05; * p¡0.1.

Figure S5: Mediation by climatic impact worries for women/non-binary: coefficient plot

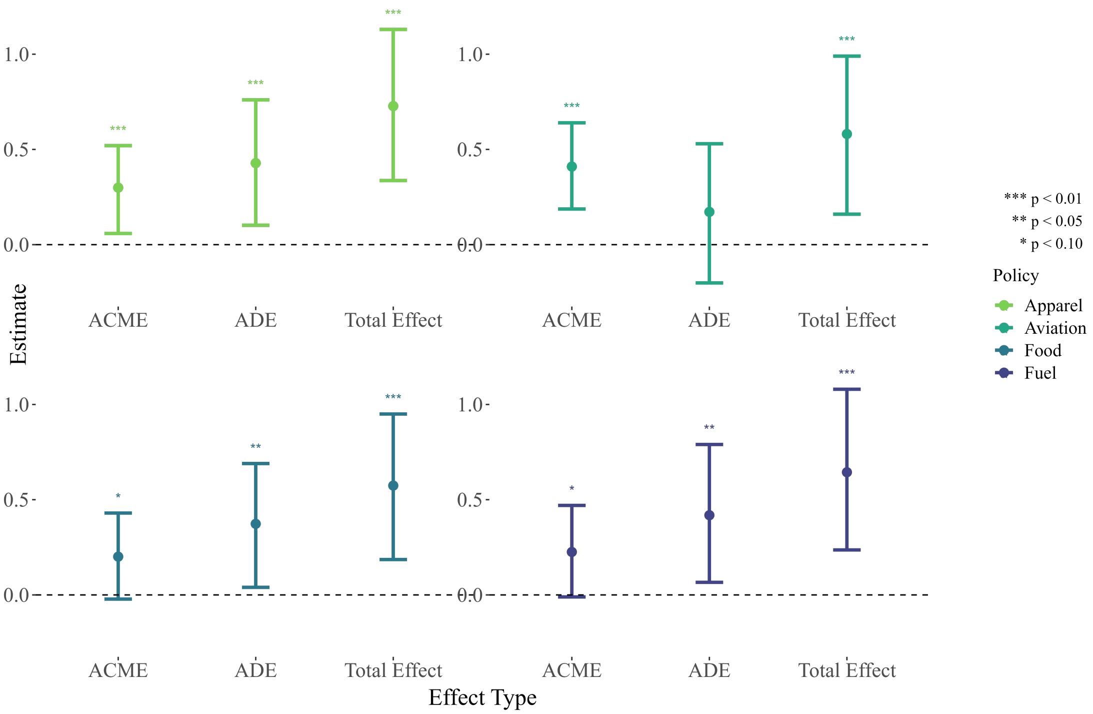

Note: The figure displays the average causal mediation (ACME) by climatic impact worries; the average direct effect (ADE); and the total effect, separately for each policy. The sample size is 793 and the error bars represent 95% confidence intervals.
